# The Value of Follow-Up Liver Stiffness Changes Measured by Virtual Touch Quantification Elastography for Predicting Recurrence of Gastroesophageal Varices after Endoscopic Injection Sclerotherapy on Cirrhotic Patients

**DOI:** 10.1155/2024/6802870

**Published:** 2024-04-25

**Authors:** Yayang Duan, Jinfei Zhang, Min Fan, Derun Kong, Chaoxue Zhang

**Affiliations:** ^1^Department of Ultrasound, The First Affiliated Hospital of Anhui Medical University, No. 218 Jixi Road, Shushan District, Hefei 230022, Anhui Province, China; ^2^Department of Ultrasound, People's Hospital of Fuyang City, No. 501 Sanqing Road, Yingzhou District, Fuyang, Anhui 236000, China; ^3^Department of Dermatology, The First Affiliated Hospital of Anhui Medical University, No. 218 Jixi Road, Shushan District, Hefei 230022, Anhui Province, China; ^4^Department of Gastroenterology, The First Affiliated Hospital of Anhui Medical University, No. 218 Jixi Road, Shushan District, Hefei 230022, Anhui Province, China

## Abstract

**Background and Aims:**

Recurrence of gastroesophageal varices (GEVs) after sclerotherapy is a public health problem. However, mass screening of recurrence of GEVs through gastroscopy is a high-cost procedure. We aim to evaluate the changes in liver stiffness (LS) over time after endoscopic injection sclerotherapy (EIS) and determine its value in predicting the recurrence of GEVs.

**Methods:**

One hundred and thirty-five patients with GEVs who underwent EIS treatment were included in this study. The patients were divided into two groups, namely, the nonrecurrence and recurrence groups, based on endoscopic findings at 6 months after discharge. LS measurements were obtained on five occasions. Repeated measure analysis of variance was employed to assess LS differences at different time points and compare them between the two groups.

**Results:**

The LS values during the 6-month postdischarge period were consistently higher than the baseline value (measured on the day of hospitalization). The recurrence group demonstrated sustained elevated LS levels throughout the 6-month follow-up period, while the nonrecurrence group showed a gradual decline in LS. The difference in LS trend between the two groups was statistically significant (*P* = 0.04). The area under the curve (AUC) values for LS differences were 0.806, with a corresponding 95% confidence interval (CI) of 0.640-0.918 and a cut-off value of 0.556, indicating their potential utility in predicting GEV recurrence.

**Conclusions:**

Longitudinal assessment of LS values in post-EIS patients can provide valuable information for predicting the recurrence of GEVs.

## 1. Introduction

Portal hypertension (PH) is a significant complication of liver disease that occurs due to increased resistance, increased blood flow, or both in the portal circulation [1, 2]. The primary reason is liver cirrhosis. This sustained elevation of portal vein pressures can give rise to various complications, such as gastroesophageal varices (GEVs), hepatic encephalopathy, ascites, or a combination of these conditions [3, 4]. Among these, the development of GEVs is widely recognized as a major consequence of PH [5], which can potentially lead to life-threatening variceal hemorrhage. In such cases, endoscopic injection sclerotherapy (EIS) is a well-established nonsurgical procedure used to prevent bleeding from GEVs. By injecting sclerosing agents, an aseptic inflammatory response is triggered in the veins and surrounding mucosal tissue, leading to the formation of a dense layer of fibrous tissue that obliterates the vessels.

Despite the widespread use of endoscopic treatment, recurrence of GEVs remains a significant challenge [6, 7]. According to the Prevention and Treatment Guidelines for Gastroesophageal Variceal Hemorrhage in Liver Cirrhosis, after complete eradication of varicose veins under endoscopy, it is recommended to undergo follow-up gastroscopy every 6 to 12 months. If varices recur, immediate endoscopic treatment should be performed. Therefore, surveillance endoscopy is necessary to monitor for variceal recurrence and determine the need for additional treatment. However, mass screening of recurrence of GEVs through gastroscopy is a high-cost procedure that can cause throat irritation, cough, nausea, bleeding, and even perforation, which can deter patients from undergoing repeat procedures. Thus, it is essential to propose a reasonable method to avoid unnecessary repeat gastroscopies.

Liver stiffness (LS) is commonly assessed using imaging techniques such as ultrasound elastography and magnetic resonance imaging elastography to stage fibrosis in clinical practice [8, 9]. Subsequent studies have demonstrated a strong association between LS and PH. For instance, Vizzutti et al. [10] established a significant correlation between LS and PH, as measured by the hepatic venous pressure gradient (HVPG), in the overall population (*r* = 0.81, *P* < 0.0001). Similarly, Lunova et al. [11] reported a good correlation between LS and PH predominantly determined by HVPG in patients with advanced liver cirrhosis. Therefore, given the connection between LS and PH, LS can predict the occurrence of GEVs. For example, a multifactor analysis conducted by Furuichi et al. [12] demonstrates the factors of LS 6 month > 19.9 kPa and splenic stiffness (SS) day 7 > 21.7 kPa were predictors of the occurrence of EGV after balloon-occluded retrograde transvenous obliteration. Abe et al. [13] indicate the combination of SS and LS and furthermore increase the diagnostic yield to detect esophageal varices. Previous literature [14, 15] also supports the notion that EIS also lead to a transient increase in HVPG and PH following the procedure, indirectly causing a rise in LS. However, whether the changes in LS after EIS can be applied to predict the recurrence of GEVs is currently unknown.

Based on this, we hypothesised that changes in LS induced by sclerotherapy may be associated with the recurrence and reperfusion of varicose veins. Therefore, the present study was performed to investigate the value of longitudinal changes in LS (*Δ*LS) after EIS measured by visual transient elastography (ViTE) for predicting the recurrence of GEVs.

## 2. Materials and Methods

### 2.1. Study Population

**Figure 1:**
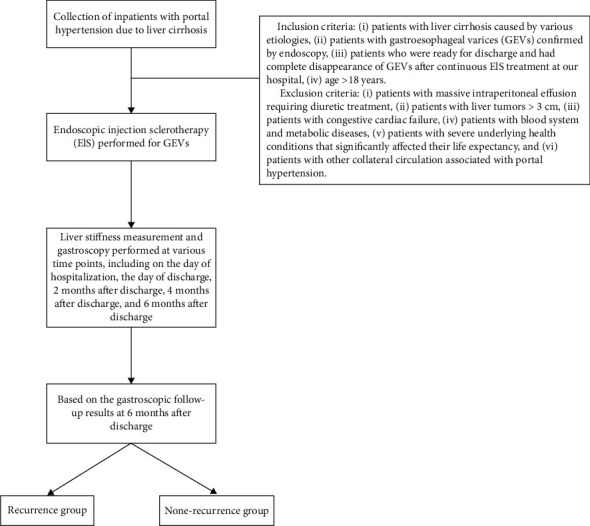
Flow chart of the study.

This study adheres to the *STROBE* guidelines. From October 2019 to December 2022, consecutive patients who underwent EIS for GEVs were recruited. The inclusion criteria were as follows: (i) patients with liver cirrhosis caused by various etiologies, (ii) patients with GEVs confirmed by endoscopy, (iii) patients who were ready for discharge and had complete disappearance of GEVs after continuous EIS at our hospital, (iv) age > 18 years, and (v) *complete medical* records. Exclusion criteria included the following: (i) patients with massive intraperitoneal effusion requiring diuretic treatment, (ii) patients with liver tumors > 3 cm, (iii) patients with congestive cardiac failure, (iv) patients with blood system and metabolic diseases, (v) patients with severe underlying health conditions that significantly affected their life expectancy, and (vi) patients with other collateral circulation associated with PH. The flow chart of patients enrolled in the study is shown in Figure 1. The training cohort contained 96 patients enrolled from October 2019 to January 2022. The validation cohort included 39 patients enrolled between February 2022 and December 2022.

### 2.2. Baseline Characteristics

Baseline clinical data, including age, gender, body mass index (BMI), etiology of the liver disease, levels of albumin (ALB), total bilirubin (TB), alanine aminotransferase (ALT), aspartate aminotransferase (AST), r-glutamyl transferase (GGT), alkaline phosphatase (ALP), creatinine, platelets (PLT), and international normalized ratio (INR), were extracted from the medical records.

Prior to EIS, blood samples were collected from all participants in the early morning for biochemical examination. Furthermore, prognostic scores such as the Model for End-Stage Liver Disease (MELD) and Child-Pugh score were calculated for each patient.

The grading of GEVs was done according to the guidelines provided by the American Society for Gastrointestinal Endoscopy [6]. The grading system categorizes GEVs into three grades: light, medium, and severe. Light: GEVs are either linear or slightly tortuous, without red sign. Medium: GEVs are either linear or slightly tortuous, with or without red sign. Severe: GEVs are characterized by being serpentine and tortuous, with or without the presence of red signs which can also be described as beaded and nodular varices, regardless of whether red signs are present or not.

In addition to the grading system, GEVs were classified into two types based on the Sarin-typing classification system [16]: GEV1: GEV1 refers to varices that are an extension of esophageal varices. These varices extend from the gastroesophageal junction downwards for a length of 2 to 5 cm along the lesser curve of the stomach. GEV2: GEV2 varices extend beyond the gastroesophageal junction and into the fundus of the stomach.

### 2.3. Follow-Up LS Measurement

LS measurements were conducted using an ultrasound machine, specifically the Mindray-Hepatus 5 from Mindray, Shenzhen, China. The ultrasound examination involved two-dimensional imaging and ViTE. The LS measurements were performed by an ultrasound physician with 6 years of experience, following the guidelines provided by the European Federation of Societies for Ultrasound in Medicine and Biology [17]. To obtain the LS measurements, the ultrasound probe was positioned vertically in the intercostal region of the patient who was in a supine position. Care was taken to avoid any interference from the pipeline structure within the liver. The sampling frame, which is the area of interest for the elasticity measurement, was placed in the parenchyma (the functional tissue) of the right anterior lobe of the liver to measure the liver stiffness.

To ensure reliable results of LS measurements, the ViTE system is equipped with a double-loop quality control function. The quality control indicators include the probe pressure ring (P) and the motion stability ring (M-STB) command indicators. These indicators are displayed as yellow and green lights, providing intuitive feedback on the operation and quality control of the examination. LS measurements were taken five times for each patient at specific time points: on the day of hospitalization, the day of discharge, 2 months after discharge, 4 months after discharge, and 6 months after discharge. To ensure consistency and accuracy, the Q-Scan intelligent acquisition function was used to obtain LS values. Each patient's measurements were taken successfully in the same position ten times. A ratio of the interquartile range (IQR) to median (M) lower than 30% was considered a valid detection. The LS values obtained were recorded as the median (interquartile interval) (M/IQR). The *Δ*LS differences were calculated as the absolute value of the difference in LS between different time points.

### 2.4. EIS and Evaluation of Recurrence

The GIF-H260 device from Olympus was used for EIS. Treatments were performed by experienced endoscopists with over 10 years of clinical experience. The endoscopists followed the European guidelines for sclerotherapy of varices, ensuring standardized and appropriate treatment protocols were followed [18, 19]. After the patients were discharged, patients also underwent gastroscopy at the time points of 2 months, 4 months, and 6 months after to determine if there was any recurrence of GEVs. The outcome of the endoscopic examination was categorized into two groups based on the findings at 6 months after discharge. Nonrecurrence group: the endoscopic examination at 6 months after discharge showed no evidence of recurrence of varicose vessels. Recurrence group: the endoscopic examination at 6 months after discharge revealed the presence of recurrent varicose vessels.

### 2.5. Statistical Analysis

Statistical analysis was conducted with SPSS 23.0 and MedCalc 15.2 software for Windows. Variables with a normal distribution were shown as the mean ± standard deviation (SD) or the median and interquartile range (IQR) with nonnormal distribution. Differences between these variables were analyzed with *t* test for normally distributed data and a Mann–Whitney *U* test for nonnormally distributed to compare baseline characteristics for continuous variables, while a chi-squared test was used to compare the differences for categorical variables. LS changes over time were analyzed by repeated measurement analysis of variance (ANOVA). *Δ*LS at different time points were calculated.

Univariate and multivariate analyses were estimated by logistic regression analysis. Univariate factors with *P* < 0.05 were entered into the multivariate analysis. The discriminative ability of established models was assessed by area under the receiver operating characteristic (ROC) curve (AUC) analysis. A two-sided *P* value less than 0.05 of the reported level was considered statistically significant.

## 3. Results

### 3.1. Baseline Characteristics

Table 1 presents the clinical characteristics of the enrolled cohort. After excluding 5 patients identified as recurrent cases during the 2-month and 4-month follow-up endoscopic examination after discharge, 135 patients were included, with 61 (45.2%) in the recurrence group and 74 (54.8%) in the nonrecurrence group. The between-group and within-group differences were not statistically significant among these clinical characteristics.

### 3.2. Mauchly's Test of Sphericity

Mauchly's test of sphericity reveals a *P* value of < 0.01 indicating that the data does not satisfy the spherical hypothesis. Therefore, *P* value of univariate analysis of variance was referenced with Greenhouse–Geisser correction performed. Refinement statistics are shown in Table 2.

### 3.3. Follow-Up LS Changes

**Figure 2:**
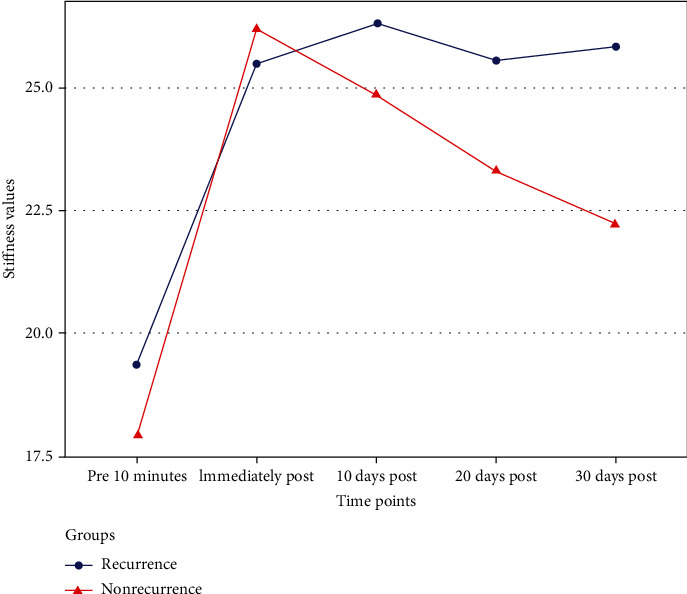
LS changes over time between the nonrecurrence and recurrence EIS groups.

There were no significant differences between the groups in terms of LS values (*P* within subjects: 0.57). However, there was a statistically significant result in *P* time (<0.01), indicating significant changes in LS over time. The *P* value of time∗groups measured the interaction effect between the timeline and groups, and it was found to be 0.04, suggesting that LS changes over time varied between the two groups.  Figure 2 showed that there was a significant increase in LS from the day of hospitalization to the day of discharge. Furthermore, the LS values at discharge, 2 months after discharge, 4 months after discharge, and 6 months after discharge were all higher compared to the LS values on the day of hospitalization. In the recurrence group, LS values remained relatively stable over time. Specifically, the average LS value was 19.35 ± 6.21 on the day of hospitalization, 25.49 ± 9.73 on the day of discharge, 26.31 ± 10.14 2 months after discharge, 25.56 ± 9.83 4 months after discharge, and 25.83 ± 9.93 6 months after discharge. In contrast, the nonrecurrence group experienced a significant setback postoperatively, with LS values decreasing from 26.18 ± 9.08 on the day of discharge to 22.22 ± 8.06 at 6 months after discharge.

### 3.4. *Δ*LS Differences Evaluation

**Figure 3:**
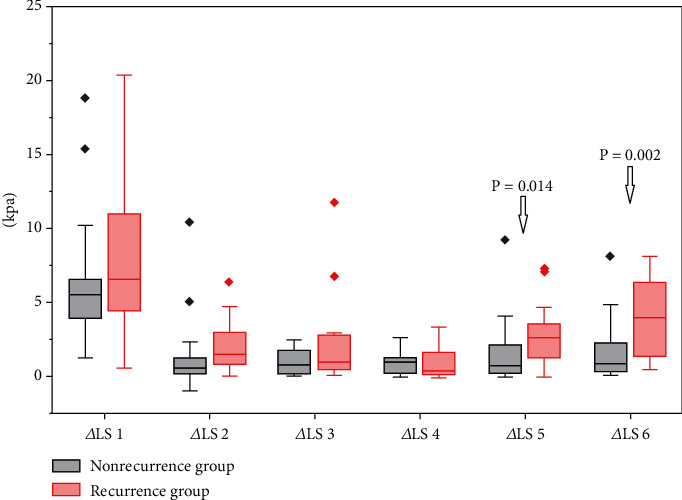
Box plots of six *Δ*LS differences for LS, categorized by nonrecurrence and recurrence group. *Δ*LS 1 (the day of hospitalization and the day of discharge), *Δ*LS 2 (the day of discharge and 2 months after discharge), *Δ*LS 3 (2 months after discharge and 4 months after discharge), *Δ*LS 4 (4 months after discharge and 6 months after discharge), *Δ*LS 5 (the day of discharge and 4 months after discharge), and *Δ*LS 6 (the day of discharge and 6 months after discharge).

To further analyze the differences between LS values at different time points, the absolute value of the difference was calculated. The Mann–Whitney *U* test showed a statistical difference between groups at two time points: the day of discharge and 4 months after discharge (*P* = 0.014) and the day of discharge and 6 months after discharge (*P* = 0.002). These results are visualized in the box plot of Figure 3.

### 3.5. Recurrence Related Characteristics

**Figure 4:**
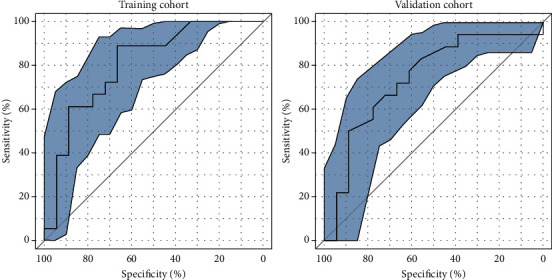
ROC curves of the *Δ*LS 6 (the day of discharge and 6 months after discharge) in the training and validation cohort.

**Figure 5:**
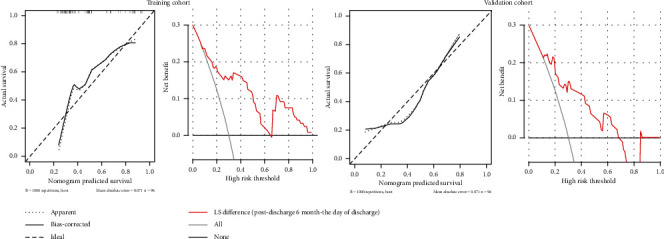
Calibration curves and DCA of the *Δ*LS model for predicting the recurrence of GEVs in the training and validation cohort.

In the training group, baseline characteristics, and *Δ*LS (the day of discharge and 4 months after discharge), *Δ*LS (the day of discharge and 6 months after discharge) were included in a univariate analysis. The results showed that only *Δ*LS (the day of discharge and 4 months after discharge) and *Δ*LS (the day of discharge and 6 months after discharge) were statistically significant in univariate analysis. Subsequently, *Δ*LS (the day of discharge and 6 months after discharge) were identified as independent cirrhosis predictors by multivariate logistic regression analysis (Table 3). The final AUC was calculated, shown in Figure 4 and Table 4. The calculated AUC values were 0.806 (95% CI of 0.640-0.918 and a cut-off value of 0.556) and 0.741 (with a corresponding 95% CI of 0.568-0.872 and a cut-off value of 0.389) in training and validation cohort, respectively. The *Δ*LS model demonstrated goodness-of-fit in predicting the recurrence of GEVs between prediction and actual endoscopic results upon bootstrapping validation. In the current study, decision curve analysis (DCA) showed that using the model to predict recurrence provides considerable benefit in the two cohorts (Figure 5).

## 4. Discussion

In this study, patients with GEVs who underwent EIS were divided into recurrence and nonrecurrence groups based on their endoscopic findings at 6 months after discharge. The main finding of the study was that there was a notable difference in the trend of LS changes over time between the two groups. Furthermore, the absolute value of LS differences at the time points of the day of discharge and 6 months after discharge was identified as a potential indicator of 6-month recurrence of GEVs. These findings suggest that monitoring LS changes over time can help predict the likelihood of GEV recurrence after EIS.

In clinical practice, the treatment outcome of EIS is closely associated with PH [17]. However, the routine assessment of PH severity relies on HVPG, which is invasive and unsuitable for long-term repeated use. Previous studies have provided evidence of a strong correlation between LS and PH. Given that the severity of PH is influenced by the blood flow in the portal vein and its collateral vessels, it follows that LS is related to the patency of the collateral vessels hardened by EIS. Therefore, monitoring changes in LS may provide insights into the recurrence of varices.

Our results indicated that there was no statistically significant difference in the degree of LS increase from the day of hospitalization to the day of discharge between the groups. The post-charge LS within 6 months were all higher than when patients were first admitted without EIS in both groups. This finding aligns with the theoretical hypothesis proposed by Avgerinos et al. [15], which suggests that a rapid dilution of the sclerosant occurs after EIS. This process leads to thrombosis and inflammation mainly in the cephalad-flowing sclerosant, a smaller amount flowing retrograde into the varix, and the remaining portion being swept into the periesophageal veins. Additionally, from an anatomical perspective, collateral circulation often occurs when portal PH appears. Since GEVs are branches of the portal vein, theoretically, any treatment to block varices will lead to changes in PH, and thus changes in LS. These factors may contribute to the increase in portal pressure following EIS. Similar studies have been reported in the literature. For example, Takuma et al. [20] assessed the values of LS and SS in the monitoring of PH and their changes to predict the exacerbation of GEVs in patients with gastric varices undergoing balloon-occluded retrograde transvenous obliteration (B-RTO). They also found that HVPG and SS increased after B-RTO, which proved useful in predicting the exacerbation of EV. Considering that B-RTO and EIS share the same physical mechanism of mechanical occlusion of varices, these findings may similarly explain the changes in LS values after EIS.

In this study, the LS values exhibited a gradual decrease in the nonrecurrent group following EIS surgery. However, despite the decrease, the LS values measured at 6 months were still higher than the pre-EIS levels. The reasons for this phenomenon may be explained as follows: Initially, sclerotherapy conducted during gastroscopy directly targets the blood vessels of GEVs, inducing vascular wall sclerosis and scar tissue formation, ultimately leading to variceal occlusion and a reduced risk of bleeding. However, even after gastroscopic sclerotherapy, their underlying liver cirrhosis remains uncured. PH resulting from liver cirrhosis does not completely resolve, although some degree of relief in PH can be achieved. Additionally, the higher the portal vein pressure level, the greater the likelihood of recurrent GEVs, and vice versa. Hence, from these perspectives, the portal vein pressure levels were consistently lower in the nonrecurrence group compared to the recurrence group, manifesting as a progressive decline in LS values throughout the six-month follow-up period. From another perspective, in terms of treatment response, the stimulating effect of the EIS can lead to a temporary increase in portal vein pressure. However, over time and with the treatment's efficacy, the portal vein pressure gradually decreases. Regarding hemodynamic changes, EIS could alter local hemodynamics, causing a transient rise in portal vein pressure. Subsequently, hemodynamics stabilize, and the portal vein pressure gradually returns to normal levels. In relation to the inflammatory response, the treatment process could trigger a certain level of inflammation, resulting in a temporary elevation of portal vein pressure. As the inflammatory response diminishes, the portal vein pressure decreases again. These factors may contribute to the gradual reduction of LS. Hence, it is conjectured that perhaps the recurrence group should also experience a decrease in LS. The differing conditions of the patients in the recurrence group may cause the progression of PH to higher levels, counteracting the downward trend of LS and presenting as a stable change after EIS. Regrettably, due to budget constraints, we were only able to follow up to six months post-EIS. The changes in LS values beyond six months, whether they are higher or lower than pretreatment levels, and the unknown factors influencing further decrease or increase, require additional research. This may suggest that for patients who receive EIS, although GEVs are improved, the high portal vein pressure levels still indicate a risk of future recurrence. Therefore, long-term follow-up and treatment to maintain disease stability and prevent complications are needed. This forms the basis for regular follow-up as recommended by guidelines and literature.

The persistent high LS values in the recurrence group after EIS may be attributed to the following explanation. As endoscopic treatment can only eliminate varicose veins and cannot resolve the underlying problem of PH in patients, although the recurrence of GEVs may share a part of the portal vein blood flow and pressure, the pressure reduction from the shunting is likely minimal compared to the high level of portal vein pressure. Therefore, considering the continuous presence of PH and its role as a risk factor for GEV recurrence, the LS values in the recurrence group may be maintained at a relatively high and stable level during the 6-month follow-up period. Hence, differences in *Δ*LS (the day of discharge and 6 months after discharge) may be a means for clinicians to be vigilant about the recurrence of GEVs within 6 months after EIS.

Currently, studies evaluating changes in LS and SS values after treating GEVs, whether through EIS or EVL, mostly focus on comparing stiffness values between two time points, typically preoperative and postoperative. In contrast, our study had a longer duration of follow-up, including measurements on the day of hospitalization, the day of discharge, 2 months after discharge, 4 months after discharge, and 6 months after discharge. This allowed us to observe and compare the trends over time in stiffness values between the two groups, which provides a more comprehensive and intuitive understanding of the changes in stiffness values after treatment for GEVs. Additionally, although the predictive efficacy of *Δ*LS (the day of discharge and 4 months after discharge) for a 6-month recurrence is lower than that of *Δ*LS (the day of discharge and 6 months after discharge), it may have a more significant clinical significance due to its ability to provide earlier indications.

Some limitations of the current study should be acknowledged. First, the small sample size in the study may limit the statistical power and exaggerate the results. To control for confounding factors, larger sample sizes would be preferable in future studies. Second, the accuracy of LS in reflecting PH may be poorer compared to SS [21–23]. As SS was not measured in our study, the clinical benefits of our results may be limited. Future studies could consider measuring and comparing both LS and SS to better understand their potential value. Third, our study only investigated the possibility of recurrence within 6 months, and the LS values of patients who experienced short-term recurrence were not included or thoroughly followed up. Therefore, our results may have some bias in terms of the LS value changes associated with short-term recurrence.

## 5. Conclusions

For GEVs receiving EIS, follow-up LS may be able to predict GEVs recurrence 6 months after discharge, in order to screen high-risk recurrence patients for gastroscopy examination, thereby reducing gastroscopy as a means of large-scale high-cost screening to a certain extent.

## Figures and Tables

**Table 1:** Baseline clinical characteristics of patients in the nonrecurrence and recurrence groups.

Characteristic	Training cohort (*n* = 96)		*P*	Validation cohort (*n* = 39)		*P*	*P ^a^*
	Nonrecurrence (*n* = 50)	Recurrence (*n* = 46)		Nonrecurrence (*n* = 24)	Recurrence (*n* = 15)		
Age (years)	56.56 ± 12.22	53.16 ± 11.60	0.436	50.04 ± 10.29	55.93 ± 12.01	0.111	0.635
Gender, no. (%)			0.510			0.217	0.626
Female	24 (48.0)	19 (41.0)		8 (33.33)	8 (53.33)		
Male	26 (52.0)	27 (59.0)		16 (66.67)	8 (53.33)		
BMI	23.08 ± 1.68	22.40 ± 2.79	0.403	22.33 ± 1.95	23.14 ± 2.57	0.268	0.392
Etiology			0.621			0.075	0.903
HBsAg	20 (40.0)	23 (50.0)		12 (50.00)	0 (0.00)		
Alcoholic	11 (22.0)	9 (20.1)		8 (33.33)	2 (13.33)		
Autoimmune	3 (6.0)	4 (8.7)		0 (0.00)	3 (20.00)		
Unknown	16 (32.0)	10 (21.2)		0 (0.00)	4 (26.67)		
Child-Pugh			0.598			0.268	0.756
A	30 (60.0)	30 (65.0)		17 (70.83)	8 (53.33)		
B	20 (40.0)	16 (35.0)		7 (29.17)	7 (46.67)		
MELD	8.00 (7.00-9.00)	8.00 (7.00-9.00)	0.385	8.00 (7.00-9.00)	8.00 (7.00-9.00)	0.953	0.634
Sarin type			0.117			0.918	0.948
GEV1	27 (61.1)	32 (36.8)		14 (58.33)	9 (60.00)		
GEV2	23 (38.9)	14 (63.2)		10 (41.67)	6 (40.00)		
Severity of GEV			0.287			0.686	0.676
Light	0 (0.0)	0 (0.0)		1 (4.17)	0 (0.00)		
Medium	5 (10.0)	2 (4.0)		4 (16.67)	2 (13.33)		
Severe	45 (90.0)	44 (96.0)		19 (79.17)	13 (86.67)		
Red sigh			0.509			0.528	0.376
Negative	1 (2.0)	2 (4.0)		3 (12.50)	3 (20.00)		
Positive	49 (98.0)	44 (96.0)		21 (87.50)	12 (80.00)		
Portal vein thrombosis			0.474			0.784	0.860
Negative	36 (72.0)	30 (65.0)		17 (70.83)	10 (66.67)		
Positive	14 (28.0)	16 (35.0)		7 (29.17)	5 (33.33)		
Ascites			0.08			0.440	0.716
Negative	19 (38.0)	10 (21.7)		11 (45.83)	5 (33.33)		
Positive	31 (62.0)	36 (78.3)		13 (54.17)	10 (66.67)		
TB (*μ*mol/L)	14.68 (11.15-17.84)	15.71 (13.34-21.39)	0.217	14.91 (10.61-18.95)	12.36 (10.79-14.88)	0.953	0.741
ALT (IU/mL)	23.5 ± 13.42	26.74 ± 13.25	0.427	26.54 ± (13.02)	22.67 ± (7.89)	0.306	0.429
AST (IU/mL)	28.94 ± 9.89	31.53 ± 11.61	0.404	23.83 ± 9.15	28.53 ± 9.43	0.131	0.178
ALP (IU/mL)	94.00 (72.75-116.25)	97.00 (79.00-133.00)	0.402	99.50 (80.00-144.75)	95.00 (75.00-108.00)	0.098	0.933
ALB	34.25 (30.65-36.90)	35.75 (33.15-39.63)	0.137	34.60 (31.40-35.10)	35.10 (32.00-39.10)	0.422	0.452
GGT (IU/mL)	29.50 (20.75-42.75)	34.00 (25.00-49.00)	0.128	30.00 (21.00-48.25)	32.00 (25.00-43.00)	0.464	0.895
Creatinine (IU/mL)	59.37 ± 12.22	65.44 ± 19.57	0.245	64.90 ± 11.53	64.57 ± 20.66	0.956	0.281
PLT (×103/*μ*L)	68.50 (53.50-135.50)	82.00 (48.00-142.00)	0.704	68.50 (46.50-89.00)	74.00 (41.00-140.00)	0.585	0.487
INR	1.14 ± 0.12	1.13 ± 0.12	0.857	1.16 ± 0.12	1.17 ± 0.14	0.975	0.865
Post beta-blockers			0.670			0.603	0.317
Not received	25 (50.0)	25 (54.3)		14 (58.3)	10 (66.7)		
Received	25 (50.0)	21 (45.7)		10 (41.7)	5 (33.3)		

Data were shown as the mean ± standard deviation (SD) for normal distribution or the median and interquartile range (IQR) with nonnormal distribution. BMI: body mass index; TB: serum total bilirubin; ALT: alanine aminotransferase; AST: aspartate aminotransferase; ALP: alkaline phosphatase; GGT: glutamyl transpeptidase; INR: international normalized ratio; ALB: albumin; PLT: blood platelet; INR: international normalized ratio. *P*^a^Comparison between training dataset and validation dataset.

**Table 2:** LS changes over time in the recurrence and nonrecurrence groups.

Outcome	The day of hospitalization	The day of discharge	2 months after discharge	4 months after discharge	6 months after discharge	Mauchly's test of sphericity	*P* time	*P* time∗groups	*P* within subjects
Nonrecurrence	19.35 ± 6.21	25.49 ± 9.72	26.31 ± 10.14	25.56 ± 9.83	25.83 ± 9.93	<0.01^∗^	<0.01^∗^	0.04^∗^	0.57
Recurrence	17.90 ± 5.53	26.19 ± 9.07	24.84 ± 9.21	23.30 ± 8.57	22.22 ± 8.06				

LS: liver stiffness. *P* time indicates differences in LS variables at each time point, *P* time∗groups (Greenhouse–Geisser correction performed) measures the interaction effect between timeline and groups, and *P* within subjects evaluates whether the two groups were significantly different from each other. ^∗^*p* value < 0.05.

**Table 3:** Results of the univariate and multivariate analyses based on the training cohort.

Characteristic	Univariate analysis		Multivariate analysis	
	OR (95% CI)	*P*	OR (95% CI)	*P*
Age, years	0.986 (0.948-1.025)	0.477	/	/
Gender	0.714 (0.282-1.811)	0.478	/	/
BMI	0.970 (0.809-1.163)	0.743	/	/
Etiology	0.996 (0.991-1.000)	0.035	/	/
MELD	0.873 (0.648-1.177)	0.375	/	/
Sarin type	0.795 (0.311-2.034)	0.633	/	/
Severity of GEV	0.834 (0.649-1.679)	0.951	/	/
Red sigh	0.365 (0.066-2.018)	0.248	/	/
Portal vein thrombosis	1.144 (0.414-3.164)	0.795	/	/
Ascites	1.818 (0.691-4.782)	0.226	/	/
ALB (IU/mL)	1.223 (1.050-1.424)	0.246	/	/
TB (IU/mL)	0.993 (0.971-1.017)	0.573	/	/
ALT (IU/mL)	1.024 (0.984-1.066)	0.245	/	/
AST (IU/mL)	1.308 (0.894-0.997)	0.437	/	/
ALP (IU/mL)	1.012 (1.000-1.025)	0.053	/	/
GGT (IU/mL)	1.019 (1.001-1.037)	0.133	/	/
Creatinine (IU/mL)	1.004 (0.972-1.036)	0.831	/	/
PLT (×103/*μ*L)	1.002 (0.995-1.009)	0.608	/	/
INR	1.364 (0.530-0.980)	0.106	/	/
Post beta-blockers	1.104 (0.643-0.870)	0.231	/	/
*Δ*LS 5	1.389 (1.058-1.825)	0.018^∗^	0.811 (0.542-1.211)	0.306
*Δ*LS 6	1.589 (1.234-2.045)	0.001^∗^	1.837 (1.243-2.715)	0.002^∗^

ALB: albumin; ALP: alkaline phosphatase; PLT: blood platelet; ALT: alanine aminotransferase; AST; aspartate aminotransferase; TB: serum total bilirubin; GGT: glutamyl transpeptidase; GLOB: globulin; PT: prothrombin time; INR: international normalized ratio; ALP: alkaline phosphatase; RBC: red blood cell; OR: odds ratio; 95% CI: 95% confidence intervals; *Δ*LS 5: the day of discharge and 4 months after discharge; *Δ*LS 6: the day of discharge and 6 months after discharge. ^∗^*p* value < 0.05.

**Table 4:** Performance of *Δ*LS for evaluating recurrence of GEVs.

Cohorts	AUC	95% CI	Cut-off value	Sensitivity (%)	Specificity (%)	LR (+)	LR (-)
Training cohort	0.806	0.640-0.918	0.556	88.89	66.67	2.67	0.17
Validation cohort	0.741	0.568-0.872	0.389	83.33	55.56	1.88	0.30

*Δ*LS: liver stiffness differences; AUC: area under curve; GEVs: gastroesophageal varices; 95% CI: 95% confidence intervals; LR (+): positive diagnostic likelihood ratio; LR (-): negative diagnostic likelihood ratio.

## Data Availability

Data supporting this research article are available from the corresponding author or first author on reasonable request.
